# Physical, Structural, Barrier, and Antifungal Characterization of Chitosan–Zein Edible Films with Added Essential Oils

**DOI:** 10.3390/ijms18112370

**Published:** 2017-11-08

**Authors:** Monserrat Escamilla-García, Georgina Calderón-Domínguez, Jorge J. Chanona-Pérez, Angélica G. Mendoza-Madrigal, Prospero Di Pierro, Blanca E. García-Almendárez, Aldo Amaro-Reyes, Carlos Regalado-González

**Affiliations:** 1 Department of Food Research and Postgraduate Studies, C.U., Autonomous University of Querétaro, Cerro de las Campanas S/N, Las Campanas, Santiago de Querétaro 76010, Mexico; moneg14@hotmail.com (M.E.-G.); blancag31@gmail.com (B.E.G.-A.); aldoamaro@gmail.com (A.A.-R.); 2 Department of Biochemical Engineering, National Polytechnic Institute, Av. Wilfrido Massieu Esq. Cda. Miguel Stampa S/N, Gustavo A. Madero, Ciudad de México 07738, Mexico; ginacaldero@hotmail.com (G.C.-D.); jorge_chanona@hotmail.com (J.J.C.-P.); 3 Faculty of Nutrition, Research Laboratory, Autonomous University of the State of Morelos, Vista Hermosa S/N, Cuernavaca 62350, Mexico; gabriela.mendoza@uaem.mx; 4 Department of Chemical Sciences, University of Naples “Federico II”, Complesso Universitario di Monte Sant’Angelo, Via Cinthia, 21, 80126 Napoli, Italy; prospero.dipierro@unina.it

**Keywords:** essential oils, edible films, raman spectroscopy, chitosan, zein, *Rhizopus*, *Penicllium*

## Abstract

Edible films (EFs) have gained great interest due to their ability to keep foods safe, maintaining their physical and organoleptic properties for a longer time. The aim of this work was to develop EFs based on a chitosan–zein mixture with three different essential oils (EOs) added: anise, orange, and cinnamon, and to characterize them to establish the relationship between their structural and physical properties. The addition of an EO into an EF significantly affected (*p* < 0.05) the a* (redness/greenness) and b* (yellowness/blueness) values of the film surface. The EFs presented a refractive index between 1.35 and 1.55, and thus are classified as transparent. The physical properties of EFs with an added EO were improved, and films that incorporated the anise EO showed significantly lower water vapor permeability (1.2 ± 0.1 g mm h^−1^ m^−2^ kPa^−1^) and high hardness (104.3 ± 3.22 MPa). EFs with an added EO were able to inhibit the growth of *Penicillium* sp. and *Rhizopus* sp. to a larger extent than without an EO. Films’ structural changes were the result of chemical interactions among amino acid side chains from zein, glucosamine from chitosan, and cinnamaldehyde, anethole, or limonene from the EOs as detected by a Raman analysis. The incorporation of an EO in the EFs’ formulation could represent an alternative use as coatings to enhance the shelf life of food products.

## 1. Introduction

The growing demand for high-quality and safe foods is leading to increased synthetic packaging materials production. However, there are environmental concerns about their use despite the benefits they provide, such as preserving and ensuring food quality [1]. Edible films (EFs) may be an alternative to reduce the use of synthetic polymers, while the addition of essential oils (EO) may improve physical and antimicrobial properties [2]. EOs have been extensively studied as natural products to provide benefits in food and human health. EOs or their components can be added directly to foods or incorporated into containers made of non-renewable materials, or biomaterials, such as polypropylene or chitosan, respectively, to be released during transport and storage [3].

Cinnamaldehyde is the major active component of the cinnamon EO (CN), and has shown a broad antimicrobial spectrum against bacteria, yeasts, and molds. Thus, CN could be used as a natural food preservative, and may be added into edible coatings to improve the shelf life and safety of food products [4]. There have been reports on the bactericidal effect of CN against *Salmonella*, *Campylobacter*, *Escherichia coli* O157:H7, and *Listeria monocytogenes*, and its antifungal activity against *Aspergillius* sp., *Penicillium* sp., and *Colletotrichum* sp. [5,6].

There is increased interest in the use of the anise EO (AS) due to its antioxidant capacity and antifungal effect against *Penicillium* sp. and *Aspergillus* sp. [7]. Anethole is the major active component of AS [8], which has been incorporated into edible coatings for dried fish and fruits preservation [9]. Limonene is the main component of the orange EO (OR), and has shown insecticidal, antibacterial, and antifungal activities. It has shown activity against *Streptococcus* sp., *Aspergillus* sp., and *Penicillium* sp. [10,11]. Limonene has many applications in both the food and cosmetic industries, but due to its lipophilic character, it exhibits poor water absorption, palatability, and oxidative deterioration [12].

The Food and Drug Administration of the U.S. lists all of the essential oils used in this work as Generally Recognized as Safe (GRAS). Water vapor permeability and the migration of atmospheric gases are dependent on many interrelated factors, including the polarity and structural features of polymers’ side chains, hydrogen bonding characteristics, the degree of branching or cross-linking, and the degree of crystallinity [13]. The spatial arrangement of atoms gives a recognizable shape that is unique to each molecule, and X-ray diffraction helps to elucidate the preferred molecular structure and the packing arrangement. Ultraviolet, infrared, and Raman spectroscopies are helpful for diagnosing the presence of functional groups’ interactions and the presence of hydrogen bonds that provide a unique spectral fingerprint that enables us to identify and detect various compounds [13,14].

The objective of this work was to evaluate: (1) the effect of the addition of the EOs cinnamon (CN), anise (AS), and orange (OR) in EFs prepared from a chitosan–zein blend (50%:50%); and (2) the relationship of the films’ structural matrix with their physical and mechanical properties. The effect of CN, AS, and OR incorporated into EFs was qualitatively tested on fungal growth inhibition.

## 2. Results and Discussion

### 2.1. Color, Transparency, and Optical Properties

The effect of the added essential oils on the transparency, chromaticity, and refractive index of the edible films prepared from a chitosan–zein 50:50% blend are presented in Table 1. Adding the EOs into the chitosan films significantly affected (*p* < 0.05) the a* (redness/greenness) and b* (yellowness/blueness) values of the films’ surface. When EFs were incorporated with OR and CN, the a* values showed 27% and 6% more negative values, indicating a redness tendency, whereas the EF with AN added showed the highest a* value among the treatments (greenness tendency). All EFs incorporating EOs increased their b* values, indicating a yellowness tendency. Polysaccharide films are usually colorless, essential oils show a slightly yellow appearance, and the incorporation of EOs into polysaccharide-based EFs have been reported to affect color and transparency [4,6].

AS showed the highest transparency value and the lowest refractive index of all of the EOs incorporated into an EF. Nevertheless, all edible films presented a refractive index between 1.35 and 1.55, and because they fall in the range 1.35–1.70 they are classified as transparent [15]. The refractive index of EFs incorporating CN or OR showed similar values compared to the EFs without an EO. However, the refractive index of the EF incorporating AS was significantly different (*p* < 0.05) from the values of the EF without an EO, and the EF with CN. Nevertheless, even small changes in the refractive index indicate that EO addition led to structural changes [16]. The visual properties of a film are an important factor for consumer acceptability.

### 2.2. Physical Properties

EF thickness (e) and filmogenic suspension density (ρ) changed with EO addition, achieving higher thickness values as compared to the control sample (Table 2). This effect can be partly related to the filmogenic suspension density, because a higher density was determined within these samples. In this regard, Bonilla et al. [17] working with basil reported that the higher density of a filmogenic suspension containing an EO was due to larger molecular contact between the chitosan’s CH groups and the oil compounds, weakening the polymer chain aggregation forces, and producing a more open matrix leading to a higher film thickness.

The film prepared without an EO tended to show higher surface roughness than the others; this could be due to the formation of Z agglomerates, resulting from hydrogen or disulfide bonding and hydrophobic interactions promoted by the pH at which the films’ suspensions were prepared. This effect was noted by Escamilla et al. [18] when working with EFs without EO addition, and was confirmed by Guo et al. [19].

The addition of EOs could reduce such interactions, decreasing agglomerates’ formation and promoting a smoother surface. The roughness (Ra, Rq) of the EF decreased with the addition of an EO, in agreement with Atarés et al. [20]. These authors worked with films made from soy protein isolate with added ginger and cinnamon EOs and reported that heating enables the integration of the EOs into the protein matrix, resulting in smoother surfaces.

The addition of EOs improved the water vapor barrier property of the chitosan-zein (CT-Z) EF, achieving permeability of 1.2, 1.5, and 1.6 g mm h^−1^ m^−2^ kPa^−1^ for AS, OR, and CN, respectively (Table 2). Aguirre et al. [21], working with whey protein isolate and the oregano EO, suggested a protein–EO interaction that immobilized the protein chain, producing a more ordered and tightly crosslinked structure, and consequently a lower permeability. On the other hand, the interaction of EO components such as ethers, ketones, and aldehydes with the OH groups of polymers increased the EFs’ hydrophobicity, thereby improving the water vapor barrier property [22].

Another study produced sodium caseinate films incorporating the cinnamon or ginger EOs, and the cinnamon EO was homogenously distributed in the protein matrix, whereas the ginger oil droplets showed agglomeration [20]. These authors concluded that structural differences linked to the oil type were the result of complex interactions taking place among lipids, proteins, and solvents. Another study reported that water vapor permeability (WVP) depends on different structural factors, such as the kind of matrix, the composition and amount of oil added, interactions with the polymers, and the hydrophilic–hydrophobic balance in the matrix [23].

Nevertheless, one report concluded that a decrease in the WVP of chitosan (CT)-based films with a lemongrass, thyme, or cinnamon EO added was due to an increase in the tortuosity factor of the water transfer within the film matrix [24]. Bonilla et al. [17] reported a decrease in the WVP of CT films with an increasing concentration of the basil EO, suggesting that the water molecules’ diffusivity decreased because of the hydrophobic nature of the EOs that predominated over the cohesion forces of the CT matrix.

#### Mechanical Properties

The addition of EOs decreased the elastic modulus and increased the hardness of the EF without an EO. All EFs incorporating an EO did not show a significant difference in the elastic modulus, whereas the AS EF exhibited the highest hardness value (Table 3). The addition of lemongrass, rosemary pepper, and basil EOs decreased the elastic modulus of a cellulose-based EF, suggesting that the interactions of EF polymers with an EO were similar to those exerted by plasticizers [25]. An EF made from fish skin gelatin with added CN, basil, plai, and lemon EOs showed a decreased elastic modulus, but a larger elongation at break due to the replacement of protein–protein interactions by EO addition in the film network [26]. This report agrees with that of Zinoviadou [26], who worked with whey protein with the oregano EO added. In this regard, the three-dimensional structure of proteins stabilized by hydrogen and disulfide bonds should be disrupted to obtain separate, entangled macromolecules to achieve plastic-like properties. The EO and other plasticizers are able to reduce the inter- and intra-molecular interactions and increase films’ flexibility depending on the oil and protein compatibility [20].

### 2.3. X-ray Diffraction

To confirm structural changes in an EF matrix, X-ray diffraction and Raman spectroscopy analyses were conducted. EFs prepared without EO addition showed two well-defined peaks, one at 7.5° and the other at 20° (2θ); however, regarding EO-containing films, two peaks, one at 2.7° and another at about 10° (2θ), can be noted.

**Figure 1:**
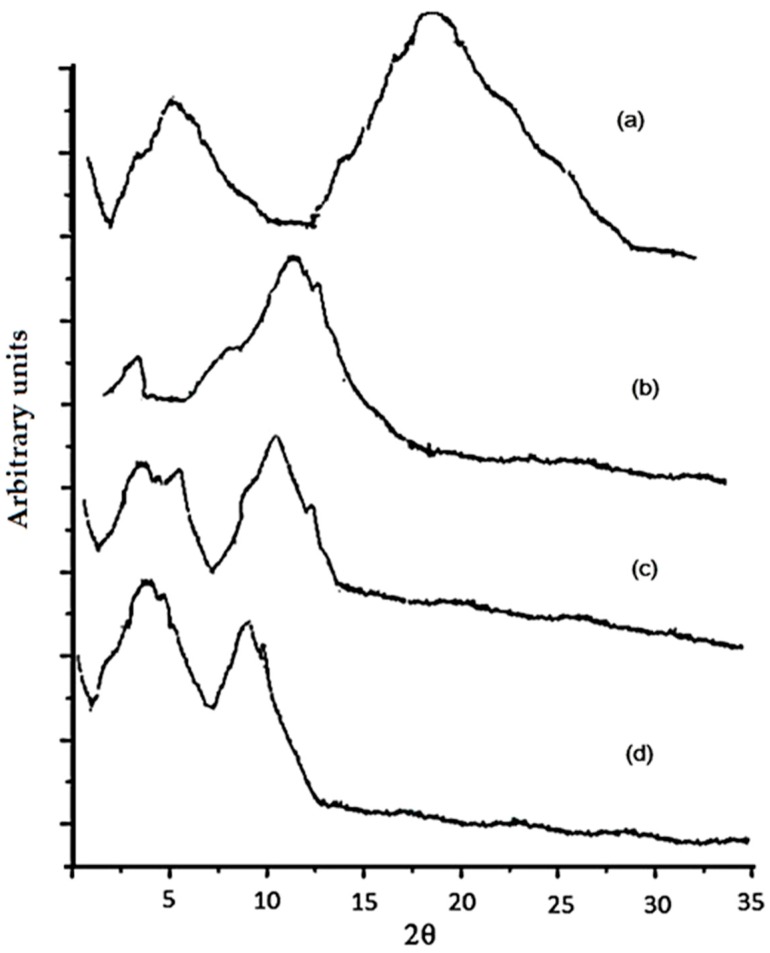
X-ray difractrograms of EF based on a mixture of zein–chitosan (**a**) Without essential oil (% *C* = 22.4 ± 0.5); (**b**) With the cinnamon (CN) essential oil (% *C* = 27 ± 0.3); (**c**) With the anise (AS) essential oil (% *C* = 32 ± 0.2); (**d**) With the orange (OR) essential oil (% *C* = 21.0 ± 0.4).

Based on Equation (14), AS addition promoted higher crystallinity than the other EOs added to the EFs, which was probably associated with higher components miscibility in the films’ matrix [27]. On the other hand, OR added to an EF barely decreased the crystallinity (% *C*: 21.0 ± 0.4 vs. 22.4 ± 0.5) (Figure 1). According to Sánchez-Gonzalez et al. [28], an EO added to an EF based on CT only increased the crystallinity up to 50%, whereas glycerol increases resulted in a lower EF crystallinity due to the higher mobility of the polymer chains [27].

Thus, it is suggested that the presence of OR leads to a decreased crystallinity of the prepared films. This phenomenon corresponds to newly formed interactions between CT and OR that slightly destroy the original crystalline structure, whereas AS and CN incorporation increased the crystallinity, suggesting that AS and CN reinforced CT films, leading to more dense crystalline domains in comparison to pure CT and CT–OR; similar results have been reported by Jahed et al. [29].

### 2.4. Raman Spectroscopy

**Figure 2:**
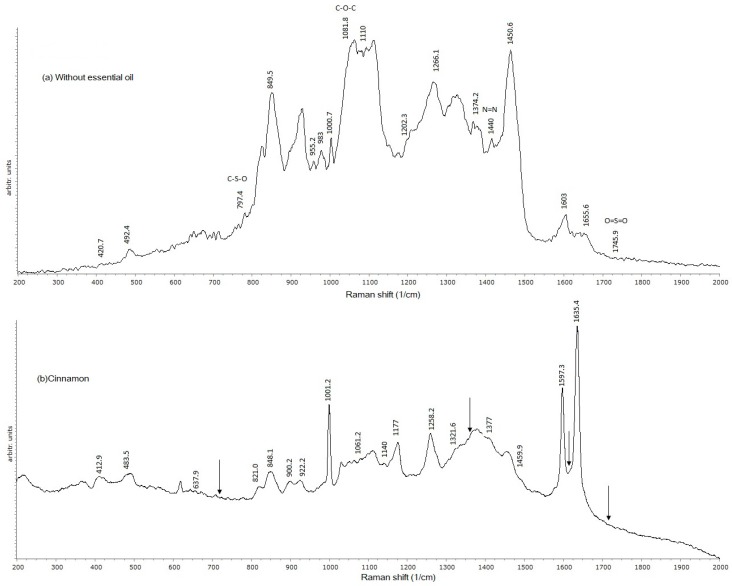
Spectrograms of EFs based on a mixture of zein–chitosan (1:1 *w*/*w*). (**a**) Without essential oil; (**b**) With cinnamon essential oil. Arrows indicate the disappearance of zein signals.

Raman spectroscopy showed that, for all EFs with an EO added, the signal at 1745 cm^−1^ disappeared (Figure 2a), which, according to Gizem-Gezer et al. [30], is a characteristic Z signal that corresponds to the functional group O=S=O. This was attributed to interactions between the protein and EO components.

The signal at 1666 cm^−1^ was associated with the partial acetylation of the NH_2_ group of CT [31]; it also disappeared in the presence of an EO.

In relation to CN edible films, cinnamaldehyde, which is comprised of a mono-substituted benzene ring, an aldehyde functional group, and a conjugated double bond, reacted with CT, promoting a nucleophilic addition reaction typical of aldehydes (Figure 2b). When analyzing the EF’s structural components, the carbonyl group (C=O) provides a reactive site for nucleophilic addition, with a Schiff base (N=C) formation.

The presence of carbonyl and amino primary groups with a free electron pair allows for the formation of a Schiff base due to the electron-deficient carbonyl carbon. This was confirmed by the disappearance of the characteristic carbonyl (C=O) signal at 1745.9 cm^−1^ when the CN EO was added, which involved a signal decrease at 998–953 cm^−1^ and at 1081–1083.7 cm^−1^, both characteristic of C–O–C bonds. This finding agrees with those reported by Gao et al. [32], who studied reactions between aldehydes and CT, nucleophilic addition being the most typical reaction.

**Figure 3:**
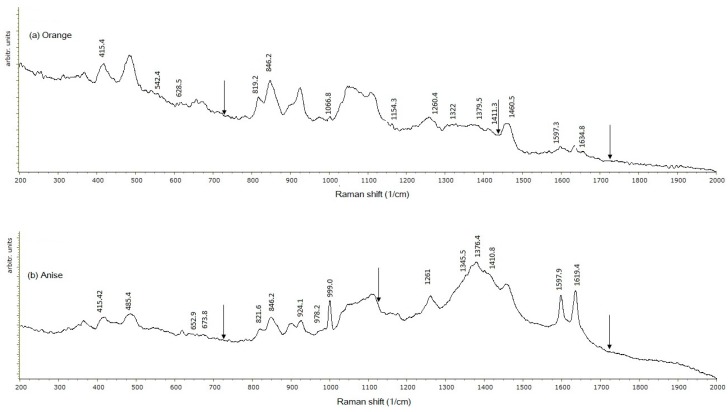
Spectrograms of EF based on a mixture of zein–chitosan (50%:50% *w*/*w*). (**a**) With orange essential oil; (**b**) With anise essential oil. Arrows indicate the disappearance of zein signals.

The spectrogram of the edible film containing the OR EO (Figure 3a) did not show characteristic signals at 1081, 1440, and 1745.9 cm^−1^ associated with the C–O–C, N=N, and O=S=O bonds, respectively. Limonene (an olefin) is the main active component of the OR EO, which in the presence of CT experiences olefin metathesis allowing for the synthesis of small and polymeric molecules by scission and the regeneration of C=C molecules [33], which were detected at 1597 cm^−1^, whereas the signals listed above disappeared.

The EF with the AS EO added showed signal disappearance at 729.74 (C–S), 1110–1150 (C–O–C), 1440 (N=N), and 1745 (C=O). These signal losses indicate the interaction of EF components (CT-Z) with the AS EO, causing bond disruption. The characteristic tyrosine signal disappeared upon the addition of the AS EO (821 cm^−1^), as well as the signal at 1745.9 cm^−1^ that is related to the Z functional group O=S=O reacting with CT [30] (Figure 3b). This implies the disruption of the protein’s secondary structure (*α*-helix) [34] so that protein structural changes due to EO addition allow for a tyrosine reaction with other film components. The reduced signal at 1619.4–1655.4 cm^−1^ indicates chemical interactions due to C=N bonds losses. The addition of each EO resulted in a signal disappearance at 729.74 cm^−1^, characteristic of the C–S aliphatic group of cysteine, suggesting the reaction of each one of the three EOs with the CT-Z EF.

From the Raman spectra, the addition of any EO confirms the X-ray analysis, since an EF’s structure was modified through the formation of a Schiff base, metathesis of CT, or disappearance of CS bonds.

### 2.5. Antifungal Activity

All EFs with an EO added showed inhibitory effects against *Rhizopus* sp. and *Penicillium* sp., whereas AS and CN showed an inhibitory effect on *Penicillium* sp., though a lower antifungal effect was observed for *Rhizopus* sp. (Table 4). The inhibitory effect of the AS EO was attributed to anethole, its main bioactive compound [8], which is effective against mycotoxigenic fungi, such as *Rhizopus* sp. and *Penicillium* sp. [7]. Among the EOs, OR showed the smallest inhibitory effect on each of the two tested fungi. The amount of each EO used to conduct the antifungal tests was 15.6 ppm, which is below the daily ingestion limit of 250 ppm [35]. Thus, higher doses may be more effective against these fungi without exceeding the permitted level. Concentrations of 100 ppm of anethole inhibited *Aspergillus flavus* and *Aspergillus parasiticus*, whereas a complete inhibition was achieved using concentrations of 100 ppm and 200 ppm, respectively [36].

Anethole targets a fungi’s mitochondrial defense system against oxidative stress [37]. The antifungal activity of the EO has been observed at different stages of a fungi’s life cycle, including at spore germination, the formation of penetrating structures, and mycelium and sporulation development [38]. Limonene is the active ingredient of OR, and it causes changes in a microbial cell membrane’s properties, increasing its fluidity, and leading to altered permeability and homeostasis loss. In addition, the EO components of OR denature enzymes responsible for germination and sporulation. A concentration of 500 ppm of OR produced a fungistatic effect on A. flavus [28].

Cinnamaldehyde is the active compound of CN, and its mechanism of action involves cellular ultrastructural changes, including organelles disappearance and solidification and the degeneration of the cell wall and cytoplasm. An inhibitory concentration of 0.5% (*v*/*v*) was found against A. niger [38].

Although EOs’ mechanism of action is not well-defined yet, their inhibitory effect has been generally associated with their hydrophobicity, which causes changes in the permeability, ion transport, and solubilization of lipid components of the cell membrane. More studies are needed to evaluate the combined effect of the tested EOs to ascertain whether there are synergistic effects against a variety of fungi.

Microbial food spoilage is one of the main problems of the food industry, and more than 50% of fruits and fruit products, are spoilt by fungi; in the baking industry, these losses represent between 1% and 3%. In addition, antifungal packaging has been proposed to extend the safety and shelf life of ready-to-eat foods [39]. Thus, active edible films may represent a good alternative for food preservation in the baking and fruit industry, and are suitable for direct food products consumption.

## 3. Materials and Methods

### 3.1. Materials

Anise (Essencefleur, Aceites y Esencias Cat. No. 98019, Ciudad de Mexico, Mexico), cinnamon (Essencefleur, Cat. No. 91041) and orange (Essencefleur, Cat. No. 10002) EOs were used. Chitosan (≥75% deacetylation, molecular weight of 375 kDa, Sigma-Aldrich, St. Louis, MO, USA), Zein (Ingredion, San Juan del Río, Qro., Mexico), *Penicillium* sp., and *Rhizopu*s sp. were acquired from the type culture collection of the Escuela Nacional de Ciencias Biológicas (ENCB, IPN, Ciudad de Mexico, Mexico). 

### 3.2. Films Preparation

Suspensions of CT (1% CT in 1 N acetic acid, *w*/*v*), and Z (1:10, Z:ethanol, *w*/*v*), were prepared by mixing in a hot plate (Barnstead, Thermolyne, MN, USA) for 15 min at 200 rpm at 90 °C; these solutions were mixed in a 1:1 ratio. Three different EFs were elaborated incorporating an EO (AS, CN, and OR) to promote ingredients solubilization and films formation; the pH was adjusted to 9.4 with 0.5 M NaOH [40]. An AS, an OR, or a CN EO was added to reach 250 ppm and was mixed for 10 min at room temperature (~24 °C). Glycerol (Sigma-Aldrich) was added at a 3:1 ratio (Z:glycerol, *w*/*w*) and stirred for 10 min for complete homogenization. Finally, 10 g of the CT-Z-EO mixtures were poured into petri dishes (90 × 15 mm) and dried in a convection oven (Terlab, Puebla, Mexico) at constant relative humidity (RH) (45%) and temperature (40 °C) for 24 h, according to Abugoch [41]. All samples were separated from the dishes and stored at constant RH (59.7%) and temperature (20 °C) until further analysis.

### 3.3. Physical Characterization

#### 3.3.1. Color, Transparency, and Optical Properties

The films’ thickness, absorption coefficient, and refractive index were determined according to Murray and Dutcher [42] with modifications. Briefly, an ellipsometer (Uvisell LT M200 AGMS, Yvon Horiba, Longiumeau, France) at a 250 to 800 nm wavelength was used. The refractive index and thickness were evaluated by the polarization changes of incident light beams (70 °C) on the surface of the film; these changes consider the ellipsometric angles of the equipment (Δ and Ψ), which in turn are related by mathematical models (polymer and amino acids) included in the software of the same company providing the values of the refractive index and thickness for the equipment. A second method was used to confirm the ellipsometer results by using a precision digital micrometer (IP54, Newton, MA, USA), that was employed to measure five different zones of the films at random locations. EF transparency and color were determined by using a colorimeter (CR-400 Chroma Meter, Konica Minolta, Tokyo, Japan) with a D65 illumination source at a 0° viewing angle, white background, and by assuming that a completely transparent film will generate the same luminosity values (L*) as those obtained from the white calibration plate (L* = 100) and that any difference will be the result of a more opaque material (L* < 100); then, lightness is considered as equal to transparency. In this experiment, the film was placed over the calibration plate and measurements were performed at five different points of the film (center and outer parts) avoiding the edges [18]. Prior to the formation of the films, samples of each of the solutions were taken (CT-Z with and without an EO) and their density was evaluated as described by ASTM D1217-12 [43]. In a pycnometer (10 mL) at constant weight, 10 mL of the filmogenic solution was poured, the weight was determined, and the density was obtained by Equation (1):

(1) $$\rho =\frac{m}{v}$$

where *ρ* is the solution density, *v* is the volume (10 mL), and *m* is the mass of 10 mL of solution.

#### 3.3.2. Atomic Force Microscopy (AFM)

AFM provides relative and absolute roughness measurements and allows investigators to obtain three-dimensional (3D) images showing the topographic characteristics of the sample. For this evaluation, an atomic force microscope (Multimode V, Veeco, CA, USA) was used, and the tapping method was applied using silicon probes (RTESP Bruker cantilevers, Santa Barbara, CA, USA) with a resonance frequency of 286–362 kHz and a spring constant of 20–80 N m^−1^. Samples of 0.5 × 0.5 cm were used and three areas of 5 × 5 µm were scanned at a speed of 1 Hz and resolution of 256 × 256 pixels. Roughness was calculated (Equations (2) and (3)), using the Nano Scope Analysis 1.20 program (Veeco) and applying a flattening process (one grade).

(2) $$Rq=\sqrt{\frac{\Sigma \left({Z_{i}}^{2}\right)}{N}}$$

(3) $$Ra=\frac{1}{N}\overset{N}{\underset{j=1}{\sum }}Z_{i}$$

where *Z_i_* is the height deviation from the mean of heights, *Z_j_* is the maximum vertical distance between the highest and lowest data points in the image prior to the planefit, *Rq* is the standard deviation of the *Z_i_* values, *Ra* is the arithmetic average of the absolute values of the surface height deviations measured from the mean plane, and *N* is the number of points in the image [44].

#### 3.3.3. Water Vapor Permeability

Water vapor permeability was evaluated using the gravimetric method ASTM E 96–80 [45] with modifications. Slight amounts of water may accumulate on the surface of hydrophilic edible films using the cup method, and the resistance of the stagnant air layer between the bottom side of the film and the surface of the saturated salt may be significant, and, if neglected, may underestimate the water vapor transmission rate (*WVTR*) [18,46]. Permeability cups with an open mouth of known area (*A*) were used. The film was sealed on top of a permeation cup, and placed in a desiccator with a saturated NaCl solution at constant temperature (25 °C) and RH (75%). The cups contained a saturated KNO_3_ solution (HR = 95.6%), and were weighed at 1 h intervals until equilibrium was reached. The slope of the weight loss (*W_S_*) versus time (*t*) plot was obtained by linear regression, and the measured *WVTR* (*WVTR_m_*) was calculated (Equation (4)).

(4) $$WVTR_{m}=\frac{W_{S}}{tA}$$

The corrected *WVTR* (*WVTRc*) was obtained following Gennadios et al. [47] using Equations (5)–(9).

(5) $$WVTR_{C}=WVTR_{m}\times \frac{P_{w1}-P_{w2}}{P_{w3}-P_{w4}}$$

(6) $$P_{w1}=P_{0}\times \frac{RH_{1}}{100}$$

(7) $$P_{w2}=P_{0}\times \frac{RH_{2}}{100}$$

(8) $$P_{w3}=P_{T}-{\left(P_{T}-P_{w1}\right)}^{\frac{WVTR\times R\times T\times h_{i}}{P_{T}\times D}}$$

(9) $$P_{w4}=P_{T}-{\left(P_{T}-P_{w2}\right)}^{\frac{WVTR\times R\times T\times h_{0}}{P_{T}\times D}}$$

where *A* is the cross-sectional area of the film, *P_w_*_1_ is the partial pressure inside the permeability cell, *P_w_*_2_ is the partial pressure inside the desiccator, *P_w_*_3_ and *P_w_*_4_ are the pressures below and above the edible film, respectively, *D* is the diffusivity of water vapor through the air (2.82 m^2^/d), *R* is the gas constant, *T* is the temperature (298 K), and *P_T_* is the atmospheric pressure (85 kPa in Querétaro, Mexico).

The water vapor permeability (*WVP*) was calculated according to Gennadios et al. [47] and Alvarado-González et al. [46] (Equation (10)).

(10) $$WVP=WVTR_{C}\frac{L}{P_{W1}-P_{W2}}$$

where *WVP* is the water vapor permeability (g mm h^−1^ m^−2^ kPa^−1^), *L* is the film thickness (mm), and *p_W_*_1_ and *p_W_*_2_ are the water vapor partial pressures (kPa) over and under the film’s surface, respectively.

#### 3.3.4. Mechanical Properties

The mechanical properties evaluated were elastic modulus and hardness. These properties were obtained by a nanoindenter (CSM, Peseux, Switzerland) measuring eight different parts of the films, including the edges and central parts. The indentation conditions were: a maximum load of 2.5 mN at loading and unloading rates of 7.5 mN/min, and a pause of 35 s using a Berkovich tip of known contact area (*Ac*). From the load-displacement curve, the maximum load (*P_max_*), maximum penetration depth (*h_max_*), and stiffness of the material (*h_S_*) were obtained. Using these parameters, values of hardness (Equation (11)) and elastic modulus were evaluated (Equations (12) and (13)) [46].

(11) $$H=\frac{P_{max}}{A\times hc}$$

(12) $$E_{r}=\frac{S\sqrt{\pi }}{2\sqrt{Ac}}$$

(13) $$E_{m}=\frac{1-v^{2}}{\frac{1}{E_{r}}-\frac{1-v^{2}}{E_{i}}}$$

where *H* (MPa) is the material hardness, *E_m_* is the elastic modulus (GPa), *E_r_* is the reduced modulus, *S* is the initial unloading stiffness in the load curve, *hc* is the difference between *h_max_* and *h_S_*, *v* is the Poisson’s ratio for polymeric samples estimated as 0.35, and *E_i_* and *v_i_* are the elastic modulus (1141 GPa) of the indenter and Poisson’s ratio (0.07), respectively [47].

### 3.4. Spectroscopic Evaluation

#### 3.4.1. X-ray Diffraction

To determine the percentage of crystallinity, an X-ray diffractometer (PANalytical XPert PRO, Almelo, Netherlands) with a Cu Kα radiation line (α = 1.5418 Å) was used. Measurements were performed in Bragg–Bretano (θ to 2θ) symmetric geometry from 3° to 35° at steps of 0.01°, a time step of 100 s, 45 kV, and 40 mA for tube power. In addition, a slot of 0.5° of divergence, a Soller slit of 0.04 rad, and a mask of 10 mm were used. For optical diffraction, a pixel (2.5°) and an ultrafast X-ray detector were used to improve the quality of the diffraction pattern [18]. The percentage crystallinity (% *C*) (Equation (14)) was evaluated considering the crystalline and amorphous peaks.

(14) $$\%\;C=\frac{I_{c}}{I_{c}+I_{a}}\times 100$$

where *I_c_* is the area of the crystalline peak, and *I_a_* is the area of the amorphous peak.

#### 3.4.2. Raman Spectroscopy

To study the chemical interactions of an EF’s components, Raman spectroscopy was used. This is a high resolution photonic technique that provides chemical and structural information of organic or inorganic compounds by plotting the intensity of the emitted signal relative to the wave number where it is emitted, resulting in the identification of functional groups from the characteristic spectral patterns of the samples [48]. The films were prepared on top of a glass slide, which contained 5-mm thick glass frames along the slide’s perimeter, with the purpose of preventing sample spillage. Five milliliters of the sample solution was poured on the slide, and then the samples were dried and stored as directed in Section 3.2. The analysis was carried out using a Raman spectrophotometer (Raman Olympus BX41, Horiba, Edison, NJ, USA) coupled to an Olympus BX 41 microscope with a 50×, N.A. 0.55 objective. Film samples were irradiated with a 735 nm emission laser, with diffraction limited to 702 nm. The spectral resolution was 0.16 cm^−1^ at a 633 nm excitation wavelength, with a 1800 g/mm and charged coupled device detector, which showed enhanced quantum efficiency in the 450–950 nm spectral range. The confocal hole and entrance slit of the monochromator were fixed at 400 µm. Readings were carried out in a spectral interval of 400 to 2000 cm^−1^. The Spekwin 32 software (developer: F. Mengef, Oberstdorf, Germany) was used for data treatment.

### 3.5. Microbiological Analysis

Microbiological analysis was performed following Boonruang [49], with modifications. Fungi bspores were taken from a stock culture and incubated in PDA plates at 30 °C for 15 day to enhance sporulation. Two milliliters of sterile 0.05% (*w*/*v*) Tween 80 was added to each plate surface, and the spore suspensions were collected, followed by an adjustment to 1 × 10^7^ spores/mL. For the agar disc diffusion method, edible films were cut (0.5 cm diameter) and PDA plates were inoculated by smearing 100 µL of *Penicillium* sp. or *Rhizopus* sp. spores suspension and left to dry for 60 min in a laminar flow cabinet. Three edible film discs for each fungus were sterilized by placing them in the laminar flow cabinet under UV light (260 nm) for 30 min, at a distance of 30 cm, followed by placing them on the inoculated agar surface. From preliminary Raman studies, we did not find any difference between irradiated and non-irradiated films. The plates were incubated 72 h at 30 °C, and microbial growth was determined by measuring the inhibition zone diameter.

### 3.6. Statistical Analysis

All parameters are expressed as the mean value and its corresponding standard deviation. For each analysis, three independent films were tested, and they were evaluated at least at three different points (edges and center). Data were analyzed by one-way analysis of variance (ANOVA) using the SigmaStat 3.5 software (San Jose, CA, USA). Significant differences were determined using the Tukey test, with significance at *p* < 0.05.

## 4. Conclusions

EOs added to EFs based on CT-Z produced more elastic, harder, and crystalline films with enhanced mechanical and barrier properties. Improved physical properties were the result of chemical interactions among the reactive groups of film components, such as amino acids from zein (cysteine and tyrosine), glucosamine from CT, and cinnamaldehyde, anethole, or limonene from EOs.

In particular, these EFs showed inhibitory effects against food spoilage fungi, such as *Penicillium* sp. and *Rhizopus* sp. Disrupted tyrosine bonds were found in the EF with AS added, which in addition to the disappearance of cysteine C–S bonds likely contributed to decrease the WVP. Thus, in this study, the properties of the EFs with any of the incorporated EOs tested suggest that such EFs may represent a good alternative for a variety of food applications.

## Figures and Tables

**Table 1:** Color and transparency of edible films zein–chitosan 50:50% with essential oils.

Edible Film	% T	a*	b*	η
Without oil	88.4 ± 0.7 ^a^	−1.34 ± 0.02 ^a^	12.4 ± 2.2 ^a^	1.45 ± 0.02 ^a^
Anise	72.6 ± 1.4 ^b^	−1.10 ± 0.01 ^b^	22.6 ± 1.3 ^b^	1.35 ± 0.02 ^b^
Orange	71.6 ± 1.1 ^b,c^	−1.70 ± 0.02 ^c^	27.8 ± 1.2 ^c^	1.55 ± 0.01 ^a,c^
Cinnamon	69.3 ± 1.1 ^c^	−1.42 ± 0.01 ^d^	28.8 ± 1.3 ^c^	1.50 ± 0.02 ^c^

Data are the mean ± standard deviation. Superscript letters a–d are used next to reported values, indicating that if the same letter appears in the same column, the values compared are not significantly different (*p* > 0.05). % T: % transparency; a* and b*: chromaticity; η: refractive index.

**Table 2:** Physical properties of edible film (EF) with different types of essential oils.

Edible Film	e (µm)	ρ (g cm^−3^)	WVP (g mm h^−1^ m^−2^ kPa^−1^)	Rq (nm)	Ra (nm)
Without oil	20.02 ± 1.45 ^a^	1.33 ± 0.02 ^a^	2.92 ± 0.16 ^a^	13.94 ± 0.09 ^a^	12.63 ± 0.06 ^a^
Anise	23.92 ± 0.92 ^b^	1.72 ± 0.01 ^b^	1.21 ± 0.10 ^b^	11.63 ± 0.14 ^b^	9.12 ± 0.12 ^b^
Orange	21.43 ± 0.51 ^c^	1.42 ± 0.02 ^a^	1.62 ± 0.02 ^c^	7.24 ± 0.11 ^c^	5.64 ± 0.16 ^c^
Cinnamon	22.54 ± 0.32 ^d^	1.54 ± 0.02 ^c^	1.53 ± 0.20 ^b,c^	3.62 ± 0.12 ^d^	2.84 ± 0.09 ^d^

Data are the mean ± standard deviation. Superscript letters a–d are used next to reported values, indicating that if the same letter appears in the same column, the values compared are not significantly different (*p* > 0.05). e: EF thickness; ρ: density of solution; WVP: EF water vapor permeability; Ra and Rq: EF roughness.

**Table 3:** Mechanical properties of zein–chitosan films with essential oils added.

Edible Film	Hardness (MPa)	Elastic Modulus (MPa)
Without oil	5.91 ± 0.41 ^a^	66 ± 6 ^a^
Anise	104.33 ± 3.22 ^b^	22 ± 3 ^b^
Orange	57.51 ± 2.43 ^c^	20 ± 2 ^b^
Cinnamon	25.54 ± 1.85 ^d^	24 ± 3 ^b^

Data are the mean ± standard deviation. Superscript letters a–d are used next to reported values, indicating that if the same letter appears in the same column, the values compared are not significantly different (*p* > 0.05).

**Table 4:** Inhibitory effect of EFs with different added essential oils.

Edible Film	Average Diameter (cm)	
	*Penicillium* sp.	*Rhizopus* sp.
Without essential oil	0	0
Anise essential oil	2.2 ± 0.2 ^a^	1.5 ± 0.3 ^a^
Cinnamon essential oil	1.9 ± 0.3 ^a^	1.7 ± 0.3 ^a^
Orange essential oil	0.7 ± 0.1 ^b^	0.6 ± 0.2 ^b^

Data are the mean ± standard deviation. Superscript letters a-b are used next to reported values, indicating that if the same letter appears in the same column, the values compared are not significantly different (*p* > 0.05).
